# Contributing Factors to Colorectal Cancer Screening among Chinese People: A Review of Quantitative Studies

**DOI:** 10.3390/ijerph13050506

**Published:** 2016-05-17

**Authors:** Doris Y. P. Leung, Ka Ming Chow, Sally W. S. Lo, Winnie K. W. So, Carmen W. H. Chan

**Affiliations:** The Nethersole School of Nursing, The Chinese University of Hong Kong, New Territories, Hong Kong, China; kmchow@cuhk.edu.hk (K.M.C.); sallylows@cuhk.edu.hk (S.W.S.L.); winnieso@cuhk.edu.hk (W.K.W.S.); whchan@cuhk.edu.hk (C.W.H.C.)

**Keywords:** Chinese, colorectal cancer, literature review, risk factors, screening

## Abstract

Colorectal cancer (CRC) is a major health problem in Asia. It has been reported that the Chinese are more susceptible to CRC than many other ethnic groups. Screening for CRC is a cost-effective prevention and control strategy; however, the screening rates among the Chinese are sub-optimal. We conducted a review to identify the factors associated with CRC screening participation among Chinese people. Twenty-two studies that examined the factors related to CRC screening behaviors among the Chinese were identified through five databases. Seven factors were consistently reported to influence CRC screening behaviors in at least one of the studies: socio-demographic characteristics (educational level, health insurance, and knowledge about CRC and its screening); psychological factors (perceived severity of CRC, susceptibility of having CRC, and barriers to screening); and contact with medical provider (physician recommendation). The evidence base for many of these relationships is quite limited. Furthermore, the associations of many factors, including age, gender, income, cancer worry/fear, and self-efficacy with CRC screening behaviors, were mixed or inconsistent across these studies, thereby indicating that more studies are needed in this area.

## 1. Introduction

Colorectal cancer (CRC) is a worldwide public health challenge. Moreover, it is the cancer that has the fourth-highest mortality rate, there being an estimated 694,000 people who died from it in 2012 [1]. CRC is highly treatable in its earlier stage, and, therefore, screening for CRC presents a cost-effective prevention and control strategy. The early detection and treatment of CRC among asymptomatic patients can result in a significant reduction in mortality [2,3]. Statistical simulation models using data collected from 1975 to 2006 in the USA have also shown that the decrease in CRC rates could be majorly attributed to screening rather than to a decrease in the risk factors of CRC [4].

Despite the dramatic improvements in cancer prevention and control, the CRC-related medical problems are still challenging, especially in the Asian region, as the incidence and mortality rates of CRC there have risen tremendously during the past two decades [5]. It has been reported that certain ethnic groups in Asia, including the Chinese, are more susceptible to CRC [6]. However, the CRC screening rates in the Chinese people were low in general [7], and were the lowest among racial groups in the USA, where screening for CRC is covered by insurance [8]. It seems that Chinese people tend not to take action even when they are provided with the service free of charge or at a reduced cost. Although many systematic reviews and meta-analyses were generated in the last decade [9,10,11,12,13], the subject was under-researched in the Chinese population. A recent review of factors contributing to the participation in CRC screening among Asian Americans reported that there were only seven studies carried out specifically among Chinese Americans [13]. In addition, the publicly financed healthcare system in the Asian region placed less emphasis on screening in the past decade [6,14]. For example, in Hong Kong, CRC screening is opportunistic, although a pilot program for CRC screening with faecal immunochemical test provided at a reduced cost for adults between the ages of 61 and 70 will be launched in 2016 [15]. In China, from 2008 to 2013, five demonstration sites have been established to provide opportunistic screening for CRC to adults between the ages of 40 to 74 [16] while Taiwan in 2004 and Singapore in 2011 have already implemented nation-wide mass screening programs for CRC targeting adults age 50 or above [17,18]. The idea of preventive measures in the community has emerged only in recent years, and, therefore, the general public may thus not have sufficient literacy on understanding what to screen and how the results should be interpreted. Given the large worldwide population of the Chinese, it is important to examine the contributing factors to CRC screening among the Chinese in order to develop a culturally specific health campaign that can effectively motivate the Chinese to take the recommended action for CRC screening.

The purpose of this article is to critically review the published literature on the factors associated with CRC screening practice among Chinese people. Our goal is to generate directions for future intervention development by identifying high-risk groups and modifiable characteristics that could be targeted to promote CRC screening behaviors in the Chinese population.

## 2. Methods

A comprehensive search was conducted by using the MEDLINE, CINAHL, EMBASE, PsycINFO, and Wan Fang Data databases for articles published either in English or Chinese up to November 2014. Using MeSH, the keywords “prevention”, “screening”, “Chinese”, “Asian”, “colon”, and “colorectal” independently or their combinations were researched. These keywords were similar to those used in the previous review on the factors associated with CRC screening among Chinese Americans [13]. Abstracts and titles of the articles were then reviewed for their relevancy to our study questions by using the following inclusion criteria: (1) original studies which examined the associated factors of CRC screening with a quantitative study design; (2) studies which included samples consisting of Chinese people and which also included subgroup analysis for the Chinese; and (3) studies which compared groups of screened and non-screened persons for CRC participation. We also hand-searched the reference lists of selected articles. The selected articles were evaluated by using the guideline proposed by Johannesen and LoGiudice [19]; this provides a simple assessment of the main factors affecting study quality based on eight criteria (inclusion and exclusion criteria clearly stated; minimized selection bias (*i.e.*, whether the sample was representative); response rate ≥80%; outcome well defined; outcome measured with reliable and valid instruments (*i.e.*, independent or blind outcome assessment or record linkage to database); risk factors well defined; risk factors measured with reliable and valid instruments (*i.e.*, record linkage to database or reliability and validity of the measurement tools were reported); and findings adjusted for confounders). For each item, one score was awarded if the criterion was achieved. The factors that were examined for an association with CRC screening were recorded in all the studies, and statistically significant associated factors of CRC screening then extracted from these included studies to evaluate the extent to which they were reproducible.

The estimates of association between CRC screening and contributing factors were expressed as odds ratios (ORs) with 95% confidence interval (CIs) using adjusted ORs that the studies provided. We calculated pooled estimates of the association for a particular factor when the corresponding ORs were provided in all the studies that had been examined for the relationship. Heterogeneity was assessed by *I^2^* statistics. The fixed or random effect models were adapted to calculate the pooled estimates where appropriate.

## 3. Results

A total of 3842 publications were identified and retrieved from the databases and other sources. After the removal of duplicates, scanning of the titles and abstracts yielded 36 potentially relevant papers for full-text review, 11 of which were excluded because 10 studies did not include subgroup analysis for Chinese participants and the other study did not include comparison between screeners and non-screeners. Of the 25 eligible publications, 22 studies were covered and met these criteria, with one of the studies being published in four articles [20,21,22,23]. A total of 22 publications were included for this review because one publication reported similar findings on one type of CRC screening based on a slightly different sample size [21] and another two publications from the same study aimed at investigating the potential interaction effects between factors on CRC screening [22,23]; hence, only the publication which reported the most detailed information on factors of CRC screening was used [20].

### 3.1. Study Characteristics

Appendix Table A1 summarizes the complete findings from the 22 original studies in this review. Regarding the study design, 15 studies employed a cross-sectional design, two were case-control studies, one was a prospective study, three were interventional studies, and one study was on scale validation. Among these 22 articles, nine focused on Chinese Americans, five on Hong Kong Chinese, five on Mainland Chinese, one on Taiwan Chinese, one on Singapore Chinese, and one on Chinese Canadians. Four of the studies included female participants only [24,25,26,27], and 12 recruited subjects aged 50 or older. Across all of these studies, there were differences in both the operational definitions and the types of screening tests being examined for CRC. Most studies examined the associated factors of ever screening for CRC, two studies also examined adherence to guidelines in addition to ever screening [26,27] and one study was on compliance of annual fecal immunochemical tests (FIT) [28]. The CRC screening rate was measured by using self-reports in most of the studies, four retrieved the screening information from medical records [29,30,31,32], and three counted the returned specimens [28,33,34]. The most common CRC screening in the literature was fecal occult blood testing (FOBT), followed by colonoscopy and (flexible) sigmoidoscopy, one study also reported digital rectal examination (DRE) with FOBT [35] and another included double contrast barium enema (DCBE) [36]. The sample size of the included studies varied from 100 to 5700. Only three studies were guided by a theoretical framework, namely, the Health Belief Model [37,38] and the Health Protection Model [39].

Regarding methodological quality, 17 articles achieved a quality score of five or more and five articles achieved a quality score of four or less. The most common problem is the low reliability and validity of the outcome measure because 18 out of all the studies used self-reports. The second is the non-representativeness of the sample because most of the studies (*n* = 16) employed convenience sampling, followed by the low response rate (<80%) (*n* = 13). The risk factors were not well defined (*n* = 11), and it was unclear whether they were measured with reliable and valid tools (*n* = 9); these are the other two common problems. In addition, two studies did not perform multivariate analyses to adjust for confounders [30,40].

### 3.2. Factors Associated with CRC Screening

Table 1 reports the findings regarding the statistically significant associated factors of participation in CRC screening identified in the included studies. There was a wide range of the number of factors associated with CRC screening examined in the reviewed studies, ranging from four [31,32] to more than 20 [41]. Of the 31 statistically significant associated factors identified from the studies, 18 were reported in more than one study. In two studies, however, no statistical significant factor of CRC screening was found [31,32]. Among these 18 factors, only one factor (contact with medical provider) was eligible for pooled estimate calculation.

#### 3.2.1. Socio-Demographic Factors

A total of 19 socio-demographic factors were identified from the included studies. Among them, ten factors, including gender, age, educational level, family history of cancer, smoking status, perceived health status, health insurance, monthly household income, acculturation/residence years, and knowledge, have been examined in more than one study.

Two studies reported a significant association of gender with CRC screening; however, the results were mixed. In the compliance study in Hong Kong [28], female participants reported a higher rate compliance rate with an adjusted odds ratio (adj OR) = 1.27, whereas the study in Taiwan reported that more female participants did not complete flexible sigmoidoscopy after the educational promotion with an adj OR = 2.06 [29]. No gender differences in the CRC screening were reported in the other 11 studies [20,31,32,33,34,36,37,39,41,42,43].

The results on the association of age with CRC screening were mixed in the included literature. Five studies reported a significant association between age and CRC screening, but they differed in the age groups and the referenced age group in the analysis. One study in China reported that participants ≥60 years old were less likely to have a colonoscopy with an adj OR = 0.68 [42], and a study in Taiwan reported a higher incompletion rate of flexible sigmoidoscopy among participants 21 years or above with an OR = 1.68 [33]. On the other hand, two studies reported a lower ever screening FOBT rate in the group of participants aged <55 years (adj OR = 0.46) [35] and a higher ever CRC screening rate in the group aged >40 years (adj OR = 3.83) [36]. In another study that examined the compliance of FIT among self-referred screeners aged 50 to 70 years old, participants aged 55 to 59 years reported a lower adj OR = 0.82 (0.70–0.95), whereas participants aged >65 years reported a higher compliance rate than the reference group of participants aged 50 to 54 years (adj OR = 1.54) [28]. However, 13 studies reported no age differences in CRC screening among Chinese people [20,24,25,26,27,31,32,34,37,38,39,41,43].

There was a consistent finding in two US studies regarding educational level: educational level is positively correlated with CRC screening with respect to DRE with an OR = 2.38 [35], and FOBT/colonoscopy in the past with an adj OR = 1.58 [39], respectively. However, no educational differences were observed in other studies in the USA [25,26,43], Canada, China, Hong Kong, and Singapore [20,24,27,28,36,38,41].

Three factors, namely, family history of cancer, smoking status, and perceived health status, were examined in two Hong Kong studies [20,28]. Although the family history of cancer and smoking status were reported to be significantly associated with CRC screening, the directions of relationships of these two factors with CRC screening were inconsistent across the two studies. For a family history of cancer, a negative relationship of family history of bowel cancer with compliance to FIT was observed in Wong *et al.*’s study [28], whereas a positive relationship with ever screening for CRC by colonoscopy but a non-significant relationship with ever screening by FOBT was found in So *et al.*’s study [20]. For smoking status, Wong *et al.*’s study found that non-smokers were more likely to be adherent to annual FIT (OR = 1.84) [28], whereas So *et al.*’s study reported that ex-smokers as compared to non-smokers were more likely to have screening by FOBT (adj OR = 1.45), but insignificant results were found for screening by colonoscopy [20]. For perceived health status, both studies reported insignificant results with CRC screening.

For health insurance, all three studies that included the variable in the analysis have consistently reported a positive association with the participation in CRC screening. In a Hong Kong study on adults aged 30 to 65 years old, participants who had health insurance reported a higher rate in CRC screening in the past (adj OR = 2.06) [37]. In another two studies in China, health insurance was also found to be positively associated with ever CRC screening in the past in a convenience sample of adults aged 18 years or over (excluding healthcare professionals) (adj OR = 2.0) [36], and also in a sample of adults who were at risk for CRC (adj OR = 1.84) [42]. However, health insurance was found to be non-significant in three US studies [31,39,43]. On the other hand, the three studies that examined the factor of monthly household income reported inconsistent findings [28,36,42]: one study in China found a negative and significant relationship of a monthly household income of ≥RMB4000 with self-reported CRC screening (adj OR = 0.633) in Chinese adults [36], but non-significant results were observed in another Chinese study on middle-aged adults with a high risk of having CRC [42] and the compliance study in Hong Kong [28].

Acculturation/residence years were examined in six studies [25,27,34,35,39,44], and significant results were reported in two studies, but the results thereof were mixed: one study found that fewer years of residence in the USA were associated with an increased likelihood of receiving an FOBT during the past year [39], whereas greater acculturation into Western society was associated with an increased likelihood of having an FOBT or sigmoidoscopy among women in the other study [25]. The culture value of healthcare was examined in only one study, and it was reported that women with a more Eastern view of healthcare were less likely to be adherent [26]. Language proficiency as an associated factor of CRC screening has been examined in three Western studies. A Canadian study on female participants reported that having Cantonese as a first language was significantly associated with an increased likelihood of never screening status (adj OR = 1.85) [27], but insignificant results were reported in two US studies [31,32].

More knowledge regarding CRC symptoms, risk factors and screening contributes to a higher chance of participation in CRC screening in four studies that reported effect sizes: two used random samples, and two used convenience samples. For the two studies with random samples, when compared to participants with a low level of knowledge, participants with a middle level of knowledge of CRC symptoms were more likely to have CRC screening (adj OR = 3.33), and those with a high level of knowledge of CRC risk factors (adj OR = 2.61) were more likely to have CRC screening, as was found in a Hong Kong study [37]. In a Singapore study [38], participants with higher knowledge scores were more likely to have had FOBT during the past (adj OR = 1.03). In two Chinese studies with convenience samples, those which had a high knowledge score were more likely to have CRC screening when compared to those with a low score (adj OR = 5.30) [36] and (adj OR = 5.99) [42], respectively. In addition, the case-control studies by Cai *et al.* [29] and Chen *et al.* [30], knowledge items were also shown to have positive associations with CRC screening.

The associations of CRC screening with eight demographic variables (including prior screening for any disease [38], serious disease or cancer [20], symptoms of CRC [26], cognitive impairment [24], obesity [33], exercise [41], use of complementary medicine [20], inadequate bowel preparation [33], and constipation [33]) have also been reported to be statistically significant in one of the reviewed studies.

#### 3.2.2. Psychological Factors

A total of nine psychological factors were identified from the included studies. Among them, seven factors, including perceived severity, perceived barriers, susceptibility of having CRC, perceived benefits of screening, screening willingness, cancer worry/fear, and self-efficacy, have been examined more than one study.

The perceived severity of CRC was reported to be negatively associated with CRC screening in three studies. The Hong Kong study by Sung *et al.* [37] found that, as compared with participants with a low level of severity score, those with a middle level reported a lower chance for screening (OR = 0.28), whereas there was no difference for those with a high level of severity. In the Singapore study, a weak negative association was observed between a perceived severity score and CRC screening (adj OR = 0.97) [38]. A similar finding was also reported in a validation study that used a sample of older Chinese adults in Hong Kong [40].

Another consistent finding is that a perceived barrier was negatively associated with the CRC screening which was reported in two studies: one study reported that higher levels in knowledge barriers (adj OR = 3.3) and time barriers (adj OR = 4.68) were associated with a higher chance of ever having screened for CRC [43], and the other study reported that lower rates of CRC screening were observed in the group of participants with a middle level of perceived health/psychological barriers (adj OR = 0.42) and in the group with high perceived access barriers (adj OR = 0.22) [37], respectively. However, non-significant results on measures of barriers and CRC screening were reported in the Singapore study [38].

The susceptibility of having CRC was reported to contribute to a higher chance of participation in CRC screening in two studies (OR = 2.79) among female Chinese Americans aged 50 or over [26], and adj OR = 1.50 for CRC screening in the past and adj OR = 1.26 for FOBT/sigmoidoscopy among Chinese Americans adults aged 50 or over [39]. Non-significant results for the susceptibility of having CRC on the participation rate were also observed in five reviewed studies [25,28,34,37,44].

Consistent findings of non-significant results of two variables, namely, perceived benefits of screening and screening willingness, were also reported in more than one reviewed study. Non-significant associations of perceived benefits of screening with participation in CRC screening were reported in all of the three studies [37,38,44]. The association between screening willingness and CRC screening participation was also found to be non-significant in two studies [26,36]. The results regarding the significance of the association of cancer worry/fear and self-efficacy with CRC screening were mixed. For cancer worry/fear, one study reported significant negative associations with ever screened for CRC (adj OR = 0.66) and with FOBT/sigmoidoscopy (adj OR = 0.73) [39] but non-significant results were found in another two studies [26,27]. For self-efficacy, a significant result with CRC screening in the past (adj OR = 3.61) was reported among female Chinese Canadians aged 50 or above [27], but non-significant results were obtained in another study [39].

Two psychological variables were examined in one study [20], and they were reported to be significantly associated with CRC screening. They perceived that visiting a doctor regularly is good for health and perceived that taking dietary supplements is good for health.

#### 3.2.3. Contact with Medical Provider

**Figure 1:**
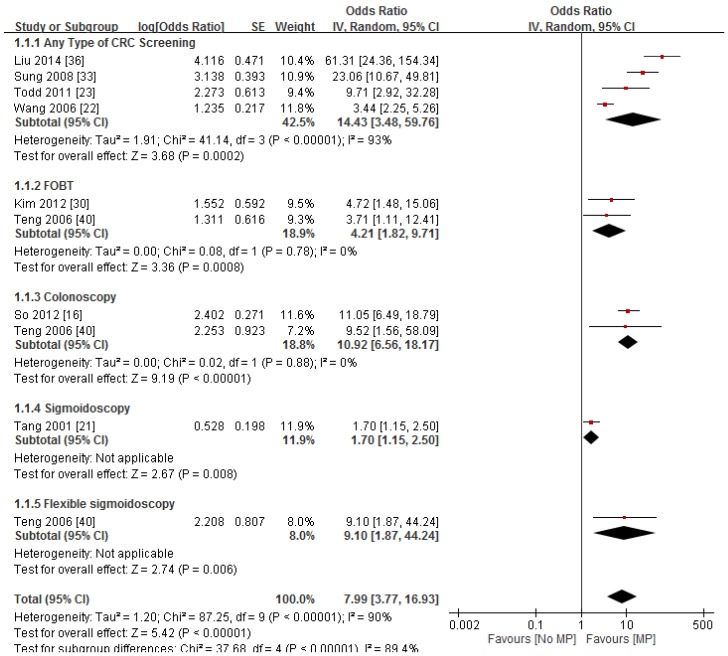
Forest plot of studies examining the association between contact with medical provider and the likelihood of CRC screening by type of screening test. MP: Contact with medical provider.

All eight studies that had examined the factor of physician recommendation found positive results with participation in CRC screening. In the Hong Kong study by Sung *et al.* [37], physician recommendation was the strongest factor associated with CRC screening and resulted in a positive direction (adj OR = 23.05). Physician recommendation was also reported as the strongest associated factor in Wang *et al.*’s study on US women aged ≥ 50 (adj OR = 3.44) [26]. Among Canadian women aged ≥ 50, those that reported who did not receive a physician recommendation were less likely to have had FOBT in the past (adj OR = 0.49) [27]. Physician recommendation was shown to be positively associated with participation in FOBT (adj OR = 3.71), flexible sigmoidoscopy (adj OR = 9.10), and colonoscopy (adj OR = 9.52), respectively, in a US study [44]. In addition, one study on community residents in Shenyang, China [41] also reported a strong association of CRC screening with recommendation for the screening (adj OR = 61.33); however, the source of the recommendation was not specified in this study, while another study in the USA found having a primary care physician was also a significant predictor (adj OR = 4.72) [34]. Another US study also reported that a lack of physician recommendation was negatively associated with FOBT screening (adj OR = 0.59) [25]. Significant results of physician recommendations were observed for colonoscopy (adj OR = 11.04) but not for FOBT in one Hong Kong study [20]. The result of the meta-analysis is depicted in the forest plot in Figure 1. The pooled ORs of the eight included studies was 7.99 (95%CI: 3.77–16.93) with a high level of heterogeneity (*I*^2^ = 89.4%). The finding of the random effect model indicates a strong positive association between contact with medical provider and the likelihood of CRC screening (pooled OR = 7.99, 95%CI = 3.77, 16.93).

Another two variables, namely, including having regular check-ups and often discussing problems and worry with family/friends, were also reported to be significant predictors of CRC screening in one study [41].

## 4. Discussion

In summary, there are a number of published studies on CRC screening among Chinese but with a wide variety in sample sizes and targeted age groups across the studies, with different types and combinations of CRC screening tests examined, and with most of the studies using self-reporting without verification. More importantly, there was a great difference in the number of factors being examined with different types and reference periods of CRC screening tests. Only a few of the reviewed studies were theoretically grounded, and many of them focused on the socio-demographic determinants of CRC screening behavior with a few psychological determinants and with contact with a medical provider included in the analysis; these factors made the comparisons across the studies very difficult. In addition, the studies varied in the operational definitions of their constructs, and only a few studies employed validated instruments to measure the factors or provided the psychometric properties of the measurement tools. In most of the included studies, they included many factors in their study that were examined with one single item, which might have affected the reliability of the measurement tools. Furthermore, the difference in the coverage of health insurance for CRC screening across countries could also explain the wide variations in the findings of the current review. Another issue regards the nature across the studies. While most of the reviewed studies were cross-sectional surveys, three studies were interventional in nature [32,33,34] in that participants were provided with the CRC screening tests after interventions. Because accessibility was reported as one component of the knowledge barrier of CRC screening [45], it is possible that the knowledge barrier of the participants in these interventional studies might have been reduced after receiving the intervention, and this in turn might have affected the findings concerning the associated factors of CRC screening. Furthermore, the results regarding the associated factors of CRC screening across countries should be interpreted with caution because of the availability of the CRC screening guidelines and screening programs. For example, there is a clear national guideline for CRC screening and population-based screening programs are available in the USA [8] but not in some Asian countries [5], and people in the Asian countries might have more barriers in having CRC screening because there was no such guideline and or population-based screening programs provided by the government.

Among the socio-demographic variables, having health insurance coverage was consistently reported to influence the likelihood of undergoing CRC screening in Hong Kong and China studies but was found to have minimal impact in US studies. This observation could be explained by the availability of the CRC screening programs and their target age groups across countries. All three US studies examining the effect of health insurance had recruited participants aged 50 or above who should be covered by insurance for the CRC screening. On the other hand, in Hong Kong and China, CRC screening is opportunistic in that people have to pay by themselves for the screening test, so it is reasonable to find that people are more likelihood to have CRC screening if the related cost is to be covered by health insurance. A higher educational level also appeared to be an important factor that positively influences the likelihood of CRC screening participation and compliance. On the other hand, knowledge of CRC symptoms and risk factors, and screening effectiveness appear to be important predictors of CRC screening. Consistent with a previous review on CRC screening behavior [9], psychological factors, including the perceived severity and susceptibility to CRC and the barriers of CRC screening, seem to have contributed to determining CRC screening, but their predictive powers were examined in only a few studies. As argued by some researchers, the inconsistent findings may be due to the cross-sectional nature of the studies or because of the mediating effects of some factors and/or effects from some important factors about CRC not included for examination [9,46] A recent study also reported that a knowledge barrier was an important associated factor of CRC screening in older Chinese adults [47]. The factor of contact with medical provider was consistently shown to influence CRC screening. The literature also suggested that the presence thereof is one of the strongest factors predicting CRC screening whereas its absence is a barrier. Our meta-analysis demonstrated a strong, consistent association between contact with medical provider and the likelihood of CRC screening among Chinese people.

In line with the findings from a review on CRC screening among Asian Americans [13], recommendation from physicians was an important factor in facilitating participation in CRC screening in the Chinese population. However, the reason for getting the tests is not known in most of the studies. While it might be possible that the Chinese tend to rely heavily on authority in making health-related decisions, it is also possible that the participants were recommended to take the test for the purpose of diagnosis of colorectal symptoms. The findings of the importance of physician recommendation in promoting CRC screening seemed however not to be translated into practice. In the two randomized controlled trials which targeted Chinese Americans or which included subgroup analysis of the Chinese [32,48], both of them tested culturally sensitive programs by providing culturally and linguistically appropriate materials delivered by a health educator, but mixed results were obtained. One study reported a significant result in screening participation [32], whereas the other reported that, in the Chinese subgroup, there was no difference in the intention to participate [48]. Possible explanations for the mixed findings include the differences in the intensity of the manipulated variables and also different outcomes were measured in the two studies. Alternatively, it is also possible that physician recommendation might also interact with other important determinants of CRC screening such as CRC-related knowledge [49]. Therefore, further studies on CRC screening that aim to disentangle the individual and interaction effects of physician recommendations and other health-related variables are needed.

In contrast to the prediction of many theories, such as the Health Belief Model [50], the current review found that perceived severity was consistently reported as being a barrier instead of a facilitator to CRC screening. The unpredicted direction of perceived severity on the CRC screening raises the concern about the ability of the theoretical models in predicting the behavior among Chinese people. Although it has been proposed that the Chinese might be more likely to try to avoid or to delay facing the adverse consequences of screening by not undergoing the tests, namely, the “ostrich” strategy, to explain this phenomenon [37,38,40], the unpredicted direction of the perceived severity may be also possibly due to the cross-sectional design of the studies in that the strength of the relationship may be underestimated or overestimated, or due to the presence of the interaction effects of severity with other psychosocial variables [49,50,51]. Experimental studies that manipulate different levels of psychosocial variables, such as perceived benefits and barriers, are needed to examine the individual and interaction effects of the perceived severity in CRC screening among Chinese people [52,53].

The current review also suggested that the susceptibility of having CRC and the benefits of screening might play only a minor role in influencing CRC screening among the Chinese participants. This might be explained, in part, by health-related cultural values. Previous studies have suggested that the Chinese tend to believe that screening is less effective as compared to diet or exercise, and hence they might feel that there is no need to have screening unless they already have symptoms or that a physician has recommended that they have CRC screening (*i.e*., benefits) [20,37,54]. In addition, Chinese people may also believe in fatalism in that they will eventually get the disease due to fate, and hence they may be less likely to take preventive measures such as adhering to CRC screening (*i.e*., susceptibility) [55]. Nevertheless, most of the studies that examined acculturation and/or language proficiency as cultural factors and their target participants were based on Chinese immigrants, reflecting that the cultural factors for CRC screening were understudied among the Chinese. Investigators should consider including cultural beliefs or values, such as cancer fatalism and perceived screening effectiveness, in future studies.

The impact of age on CRC screening among Chinese was inconsistent. This might be explained partially by the age of the target participants and the different reference age groups for comparison across the studies. Alternatively, the impact of age on CRC screening may be non-linear with an inverted U-shape rather than a linear one. According to most of the CRC screening guidelines, people should undergo screening when they reach 50 years of age. Younger people then may feel that they are not at risk of CRC and hence that there is no need for them to take action, while, on the other hand, older people may not consider screening for CRC to be necessary because they may die in the near future, and these point to lower CRC screening rates for the younger and older age groups when compared to the 50-year age group. Consequently, the creation of age groups with different cut-off points might have produced the inconsistent results in different studies. To enhance the understanding of the relationship between age and different types of CRC screening tests, investigators should consider age as a continuous variable and test for its potential non-linear relationship with CRC screening.

## 5. Conclusions

In conclusion, despite there being an increasing incidence of CRC among the Chinese, the research area regarding factors associated with CRC screening in this large group of people was understudied. Although a few demographic characteristics and personal and environmental factors of CRC screening among Chinese were identified in this review, the evidence for their strengths and directions of the relationships with CRC screening behaviors is somewhat limited. Future research should seek to broaden the evidence with the use of an experimental design that examines the individual and interaction effects of the factors that influence CRC screening. The research design should also be guided by theories that incorporate Chinese cultural beliefs and values in order to capture the cultural characteristics of this particular group. The measurement tools should be well validated and commonly used to allow better comparisons between the studies. Meta-analyses of the factors, in particular those with inconsistent results (e.g., age), should be considered when more studies in the area are available. Future studies should also focus on examining potential modifiable factors, such as self-efficacy, that could be targeted in the interventions for promoting CRC screening among Chinese people.

## Figures and Tables

**Table 1:** Associated factors of participation in CRC screening.

Study	Type of CRC Screening	Significant Factors of Screening
Cai *et al.* [29] ^1^	Data from registry of FOBT and CS	Knowledge
Chen *et al.* [30] ^1^	Data from registry of compliance (FOBT and CS)	Understanding the purpose and method of the screening
		Value CRC screening
		Knowledge about CRC screening
		Screening can improve health
Chou *et al.* [33]	FS screening at the site	For not completing of FS screening:
		Female: Adj OR = 2.06 (95%CI = 1.56, 2.73)
		Age ≥ 60: Adj OR = 1.68 (95%CI = 1.26, 2.23)
		BMI < 25: Adj OR = 1.41 (95%CI = 1.05, 1.89)
		History of constipation: Adj OR = 2.43 (95%CI = 1.04, 5.69)
		Inadequate bowel preparation: Adj OR = 1.66 (95%CI = 1.21, 2.16)
Deng *et al.* [36]	Self-reported CRC screening in the past	Age ≥ 40: Adj OR = 3.834 (95%CI = 2.657, 5.532)
		Health insurance : Adj OR = 1.996 ((5% CI = 1.426, 2.794)
		Monthly household income ≥ 4000 RMB: Adj OR = 0.633 (95%CI = 0.467, 0.858)
		High knowledge level (low as reference): Adj OR = 5.299 (95%CI = 3.415, 8.223)
Hong [42]	Self-reported CS in the past	Age ≥ 60: Adj OR = 0.682 (95%CI = 0.513, 0.916)
		Health insurance: Adj OR = 1.835 (95%CI = 1.207, 2.931)
		High knowledge (low as reference): Adj OR = 5.985 (95%CI = 3.471, 9.142)
Kim *et al.* [34]	FOBT test after educational session	Having primary-care physician: Adj OR = 4.72 (95%CI = 1.48, 15.11)
Leung *et al.* [24]	Self-reported of FOBT/endoscopy in the past two years	Cognitive impairment: Adj OR = 0.81 (95%CI = 0.66, 0.99)
Leung *et al.* [40] ^1^	Self-reported CRC screening in the past	Severity–fear
		Severity–life impact
		Psychological barriers
		Knowledge barriers
Liu [41]	Self-reported CRC screening in the past	Logistical regression with psychological factors:
		Often have health check-up (never as reference): Adj OR = 2.938 (95%CI = 1.074, 8.038);
		Have regular health check-up (never as reference): Adj OR = 6.747 (95%CI = 2.484, 18.330)
		Exercise until reach the desirable pulse rate in sometime (never as reference): Adj OR = 3.447 (95%CI = 1.503, 7.907).
		Logistical regression with environmental factors:
		Discuss problems and worry with friends/family regularly (never as reference): Adj OR = 15.281 (95%CI = 1.788, 130.613);
		Received recommendation for CRC screening: Adj OR = 61.328 (95%CI = 24.341, 154.521).
Ma *et al.* [43]	Self-reported compliance with CRC screening	For non-screening:
		Knowledge barriers: Adj OR = 3.3 (95%CI = 1.51, 7.23)
		Language barriers: Adj OR = 2.98 (95%CI = 1.2, 7.4)
		Time barriers: Adj OR = 4.68 (95%CI = 1.21, 18.11)
Ng *et al.* [38]	Self-reported FOBT in the past	Had other screening: Adj OR = 3.47 (95%CI = 1.75, 6.91)
		Influenced by family/friends: Adj OR = 2.14 (95%CI = 1.02, 4.49)
		Knowledge: Adj OR = 1.03 (95%CI = 1.01, 1.04)
		Severity: Adj OR = 0.97 (95%CI = 0.96, 0.99)
So *et al.* [20]	Self-reported ever CS; Self-reported ever FOBT	For CS:
		Male: Adj OR = 1.58 (95%CI = 1.19, 2.10)
		Family history of cancer: Adj OR = 1.43 (95%CI = 1.05, 1.95)
		Had serious disease/cancer: Adj OR = 2.62 (95%CI = 1.79, 3.83)
		Poor perceived health status: Adj OR = 1.38 (95%CI = 1.02, 1.86)
		Perceived visiting a doctor regularly is good for health: Adj OR = 2.42 (95%CI = 1.81, 3.24)
		Healthcare professional recommendation: Adj OR = 11.04 (95%CI = 6.49, 18.77)
		For FOBT:
		Had serious disease/cancer: Adj OR = 1.50 (95%CI = 1.04, 2.10)
		Ex-smoker (non-smoker as reference): Adj OR = 1.45 (95%CI = 1.05, 2.02)
		Perceived visiting a doctor regularly is good for health: Adj OR = 1.96 (95%CI = 2.49)
		Perceived taking dietary supplement is good for health: Adj OR = 1.54 (95%CI = 1.18, 2.01)
		Use of complementary medicine: Adj OR = 1.54 (95%CI = 1.18, 2.01)
Sun *et al.* [39]	Self-reported FOBT in the past 12 months; Self-reported FOBT plus SC in the past; Self-reported FOBT or SC in the past	For ever screener *vs.* non-screener:
		Years of residency: Adj OR = 0.545 (95%CI = 0.042, 0.045)
		Worry/fear: Adj OR = 0.658 (95%CI = 0.549, 0.788)
		Susceptibility: Adj OR = 1.502 (95%CI = 1.309, 1.724)
		For FOBT plus SC:
		Education year: Adj OR = 1.580 (95%CI = 1.015, 2.459)
		Worry/fear: Adj OR = 0.727 (95%CI = 0.629, 0.868)
		Susceptibility: Adj OR = 1.264 (95%CI = 1.127, 1.418)
Sung *et al.* [37]	Self-reported CRC screening uptake in the past	Middle knowledge of CRC symptoms level (low as reference): Adj OR = 3.33 (95%CI = 1.22, 9.11)
		High knowledge of CRC risk factors level (low as reference): Adj OR = 2.61 (95%CI = 1.18, 5.88)
		Middle perceived severity of CRC level (low as reference): Adj OR = 0.28 (95%CI = 0.13, 0.65)
		Middle health/psychological barriers level (low as reference): Adj OR = 0.42 (95%CI = 0.21, 0.85)
		High access barrier level (low as reference): Adj OR = 0.22 (95%CI = 0.06, 0.85)
		Health insurance: Adj OR = 2.06 (95%CI = 1.01, 4.19)
		Physician recommendation: Adj OR = 23.05 (95%CI = 10.66, 51.80)
Tang *et al.* [25]	Self-reported FOBT in the past; Self-reported sigmoidoscopy in the past	For FOBT:
		Acculturation: Adj OR = 5.54 (95%CI = 1.85, 16.60)
		For sigmoidoscopy:
		Acculturation: Adj OR = 8.70 (95%CI = 2.07, 36.55)
		Lack of physician recommendation: Adj OR = 0.59 (95%CI = 0.40, 0.89)
Teng *et al.* [44]	Self-reported FOBT in the past; Self-reported FS in the past; Self-reported CS in the past	For FOBT:
		Physician recommendation: Adj OR = 3.71 (95%CI = 1.11, 12.46)
		For FS:
		Physician recommendation: Adj OR = 9.10 (95%CI = 1.87, 44.21)
		For CS:
		Physician recommendation: Adj OR = 9.52 (95%CI = 1.56, 58.82)
Todd *et al.* [27]	Self-reported CRC screening	No physician recommendation: Adj OR = 0.103 (95%CI = 0.031, 0.349)
		Cantonese as 1st language: Adj OR = 1.85 (95%CI = 0.055, 0.628)
		Self-efficacy: Adj OR = 3.613 (95%CI = 1.179, 11.070)
Tu *et al.* [32]	Medical record of FOBT: 48.5%	No significant factors other than the intervention
Wang *et al.* [26]	Self-reported CRC screening according to US guideline	Physician recommendation: Adj OR = 3.44 (95%CI = 2.25, 5.28)
		Symptoms: Adj OR = 1.74 (95%CI = 1.10, 2.73)
		Thoughts about getting CRC: Adj OR = 2.79 (95%CI = 1.63, 4.77)
		Cultural views: Adj OR = 0.97 (95%CI = 0.95, 0.99)
Wong *et al.* [28]	Compliance of annual FIT checked by returned specimens	Female: Adj OR = 1.27 (95%CI = 1.11, 1.45)
		Age 55–59 (50–54 as reference): Adj OR = 0.82 (95%CI = 0.70, 0.95)
		Age 65–70 (50–54 as reference): Adj OR = 1.54 (95%CI = 1.26, 1.89)
		Non-smoking: Adj OR = 1.84 (95%CI = 1.43, 2.37)
		Family history of bowel cancer
		1st degree relative: Adj OR = 0.74 (95%CI = 0.61, 0.89)
		2nd degree relative: Adj OR = 0.78 (95%CI = 0.65, 0.94)
Yip *et al.* [31]	Medical record of CRC screening	No significant factor
Yu *et al.* [35]	Self-reported DRE in the past; Self-reported FOBT in the past	For DRE:
		Education ≥ 12 years: Adj OR = 2.38 (95%CI = 1.47, 3.84)
		For FOBT:
		Age < 55: Adj OR = 0.46 (95%CI = 0.29, 0.72)

^1^ Estimates of effect sizes of the factors were not available in the study.

## Abbreviations

Adj OR	Adjusted odds ratio
CRC	Colorectal cancer
FOBT	Fecal occult blood testing
FIT	Fecal immunochemical test
CS	Colonoscopy
FS	Flexible sigmoidoscopy
DRE	Digital rectal examination

## Appendix

**Table A1:** Summary of studies included in review.

First Author	Year	Country	Study Design	Sample Size	Method of Ascertainment	Selection Criteria of Participants	Effect Size	Variables Adjusted for in the Analysis	Quality Score
Todd *et al.* [27]	2011	Canada	Cross-sectional	103	Self-reported CRC screening: 75%	Women aged ≥50 years	No physician recommendation: Adj OR = 0.103 (95%CI = 0.031, 0.349); Cantonese as 19 language: Adj OR = 1.85 (95%CI = 0.055, 0.628); Self-efficacy: Adj OR = 3.613 (95%CI = 1.179, 11.070).	Years in Canada; Health literacy.	5
Cai *et al.* [29] *	2009	China	Case-control study	463	Data from registry of FOBT and CS	Adults aged 40–74 years	Knowledge	Age; Gender; Occupation; Gender by Occupation; Annual personal income.	4
Chen *et al.* [30] *	2010	China	Case-control study	453	Data from registry of compliance (FOBT and CS)	Adults aged 40–74 years	Understanding the purpose and method of the screening Value CRC screening Knowledge about CRC screening Screening can improve health	No multivariate analysis was done	3
Deng *et al.* [36]	2011	China	Cross-sectional	1001	Self-reported CRC screening in the past: 22.5%	Adults aged over 18 years excluding health care professionals	Age ≥ 40: Adj OR = 3.834 (95%CI = 2.657, 5.532) Health insurance : Adj OR = 1.996 (95%CI = 1.426, 2.794) Monthly household income ≥4000 RMB: Adj OR = 0.633 (95%CI = 0.467, 0.858) High knowledge level (low as reference): Adj OR = 5.299 (95%CI = 3.415, 8.223)	Gender; Educational level.	5
Hong [42]	2012	China	Cross-sectional	1944	Self-reported CS in the past: 24.5%	High CRC risk adults aged ≥ 40 years	Age ≥ 60: Adj OR = 0.682 (95%CI = 0.513, 0.916) Health insurance: Adj OR = 1.835 (95%CI = 1.207, 2.931) High knowledge (low as reference): Adj OR = 5.985 (95%CI = 3.471, 9.142)	Gender; Educational level; Monthly household income.	5
Liu [41]	2014	China	Cross-sectional	600	Self-reported CRC screening in the past: 22%	Adults aged 40–75 years	Logistical regression with psychological factors: Often have health checkup (never as reference): Adj OR = 2.938 (95%CI = 1.074, 8.038); Have regular health checkup (never as reference): Adj OR = 6.747 (95%CI = 2.484, 18.330) Exercise until reach the desirable pulse rate in sometime (never as reference): Adj OR = 3.447 (95%CI = 1.503, 7.907). Logistical regression with environmental factors: Discuss problems and worry with friends/family regularly (never as reference): Adj OR = 15.281 (95%CI = 1.788, 130.613); Received recommendation for CRC screening: Adj OR = 61.328 (95%CI = 24.341, 154.521).	For psychological factors: Report symptoms; Seeking health information via newspapers or TV; Ask if having difficulties in understanding health advice; Proactively seeking health advice; Attend health talk; Set exercise plan; Join physical exercise programs; Have physical exercise at least 3 times per week; Have training actively in daily life; Measure pulse during exercise; pay attention to nutrition information; release pressure; A balance between work and entertainment. For environmental factors: Spend time with close friends; satisfy the needs of close friends; Number of close friends who could provide help; Relationship with colleagues; Support from siblings; Support from other family members.	6
Leung *et al.* [24]	2012	Hong Kong	Cross-sectional	1533	Self-reported of FOBT/endoscopy in the past 2 years: 10.8%	Women aged ≥ 60 years Long-term care service applicants	Cognitive impairment: Adj OR = 0.81 (95%CI = 0.66, 0.99)	Number of chronic diseases	6
Leung *et al.* [40] *	2014	Hong Kong	Scale validation	219	Self-reported CRC screening in the past: 24.4%	Adults ≥ 60 years	Severity-fear Severity-life impact Psychological barriers Knowledge barriers	No multivariate analysis was done.	4
So *et al.* [20]	2012	Hong Kong	Cross-sectional	2004	Self-reported ever CS: 19% Self-reported ever FOBT: 12%	Random sample 50–75 years	For CS: Male: Adj OR = 1.58 (95%CI = 1.19, 2.10) Family history of cancer: Adj OR = 1.43 (95%CI = 1.05, 1.95) Had serious disease/cancer: Adj OR = 2.62 (95%CI = 1.79, 3.83) Poor perceived health status: Adj OR = 1.38 (95%CI = 1.02, 1.86) Perceived visiting a doctor regularly is good for health: Adj OR = 2.42 (95%CI = 1.81, 3.24) Health care professional recommendation: Adj OR = 11.04 (95%CI = 6.49, 18.77) For FOBT: Had serious disease/cancer: Adj OR = 1.50 (95%CI = 1.04, 2.10) Ex-smoker (non-smoker as reference): Adj OR = 1.45 (95%CI = 1.05, 2.02) Perceived visiting a doctor regularly is good for health: Adj OR = 1.96 (95%CI = 2.49) Taking dietary supplement is good for health: Adj OR = 1.54 (95%CI = 1.18, 2.01) Use of complementary medicine: Adj OR = 1.54 (95%CI = 1.18, 2.01)	For CS: Age; Health status; Smoking status; Perceived maintaining a healthy diet is good for health; Perceived visiting a Chinese herbalist regularly is good for health; Perceived taking dietary supplements is good for health. For FOBT: Educational level; Family history of cancer; Health status; Perceived health status; Perceived doing exercise is good for health; Perceived maintaining a healthy diet is good for health; Perceived visiting a Chinese herbalist regularly is good for health.	4
Sung *et al.* [37]	2008	Hong Kong	Cross-sectional	1004	Self-reported CRC screening uptake in the past: 9.9%	Random sample of Adults aged 30–65 years	Middle knowledge of CRC symptoms level (low as reference): Adj OR = 3.33 (95%CI = 1.22, 9.11) High knowledge of CRC risk factors level (low as reference): Adj OR = 2.61 (95%CI = 1.18, 5.88) Middle perceived severity of CRC level (low as reference): Adj OR = 0.28 (95%CI = 0.13, 0.65) Middle health/psychological barriers level (low as reference): Adj OR = 0.42 (95%CI = 0.21, 0.85) High access barrier level (low as reference): Adj OR = 0.22 (95%CI = 0.06, 0.85) Health insurance: Adj OR = 2.06 (95%CI = 1.01, 4.19) Physician recommendation: Adj OR = 23.05 (95%CI = 10.66, 51.80)	All variables are significant in the multivariate analysis.	5
Wong *et al.* [28]	2013	Hong Kong	Cohort	5700	Compliance of annual FIT checked by returned specimens: Year 1: 95.1% Year 2: 79.9% Year 3: 66.2% Year 4: 68.4%	Previous self-referred screeners aged 50–70 years	Female: Adj OR = 1.27 (95%CI = 1.11, 1.45) Age 55–59 (50-54 as reference): Adj OR = 0.82 (95%CI = 0.70, 0.95) Age 65–70 (50-54 as reference): Adj OR = 1.54 (95%CI = 1.26, 1.89) Non-smoking: Adj OR = 1.84 (95%CI = 1.43, 2.37) Family history of bowl cancer 1st degree relative: Adj OR = 0.74 (95%CI = 0.61, 0.89) 2nd degree relative: Adj OR = 0.78 (95%CI = 0.65, 0.94)	Monthly household income; Educational level; Marital status; Occupation; Self-perceived health status; Self-perceived risk.	6
Ng *et al.* [38]	2007	Singapore	Cross-sectional	557	Self-reported FOBT in the past: 26.5%	Random sample of adults aged ≥ 50 years	Had other screening: Adj OR = 3.47 (95%CI = 1.75, 6.91) Influenced by family/friend: Adj OR = 2.14 (95%CI = 1.02, 4.49) Knowledge: Adj OR = 1.03 (95%CI = 1.01, 1.04) Severity: Adj OR = 0.97 (95%CI = 0.96, 0.99)	Age; Education; Perceived barriers; Perceived benefits.	5
Chou *et al.* [33]	2007	Taiwan	interventional	1252	FS screening at the site: 77.8%	Self-referred adults aged 21–87 years	For incompletion of FS screening: Female: Adj OR = 2.06 (95%CI = 1.56, 2.73) Age ≥ 60: Adj OR = 1.68 (95%CI = 1.26, 2.23) BMI < 25: Adj OR = 1.41 (95%CI = 1.05, 1.89) History of constipation: Adj OR = 2.43 (95%CI = 1.04, 5.69) Inadequate bowel preparation: Adj OR = 1.66 (95%CI = 1.21, 2.16)	All variables are significant in the multivariate analysis.	6
Kim *et al.* [34]	2012	USA	Interventional	113	FOBT test after educational session	Adults aged ≥ 50 years	Having primary-care physician: Adj OR = 4.72 (95%CI = 1.48, 15.11)	Years in US, Age; Gender.	5
Ma *et al.* [43]	2012	USA	Cross-sectional	311	Self-reported compliance to CRC screening: 34.7%	Random sample of adults aged ≥ 50 years	For non-screening: Knowledge barriers: Adj OR = 3.3 (95%CI = 1.51, 7.23) Language barriers: Adj OR = 2.98 (95%CI = 1.2, 7.4) Time barriers: Adj OR = 4.68 (95%CI = 1.21, 18.11)	Psychological barrier; Insurance.	7
Sun *et al.* [39]	2004	USA	Cross-sectional	203	Self-reported FOBT in the past 12 month: 15.8% Self-reported FOBT plus SC in the past: 22.2% Self-reported FOBT or SC in the past: 37.9%	Adults aged ≥ 50 years	For ever screener vs non-screener: Years of residency: Adj OR = 0.545 (95%CI = 0.042, 0.045) Worry/fear: Adj OR = 0.658 (95%CI = 0.549, 0.788) Susceptibility: Adj OR = 1.502 (95%CI = 1.309, 1.724) For FOBT plus SC: Education year: Adj OR = 1.580 (95%CI = 1.015, 2.459) Worry/fear: Adj OR = 0.727 (95%CI = 0.629, 0.868) Susceptibility: Adj OR = 1.264 (95%CI = 1.127, 1.418)	For ever screener vs non-screener: Education; Family history of CRC; Self-efficacy; Social influence; Efficacy of screening; Intention. For FOBT plus SC: Years of residency in US, Self-efficacy; Social influence; Efficacy of screening; Intention.	6
Tang *et al.* [25]	2001	USA	Cross-sectional	100	Self-reported FOBT in the past: 25% Self-reported Sigmoidoscopy in the past: 31%:	Women aged ≥ 60 years	For FOBT: Acculturation: Adj OR = 5.54 (95%CI = 1.85, 16.60) For Sigmoidoscopy: Acculturation: Adj OR = 8.70 (95%CI = 2.07, 36.55) Lack of physician recommendation: Adj OR = 0.59 (95%CI = 0.40, 0.89)	For FOBT: No other variable was controlled in the multivariate analysis. For Sigmoidoscopy: Income; modesty; use of Eastern medicine.	4
Teng *et al.* [44]	2006	USA	Cross-sectional	206	Self-reported FOBT in the past: 65.0% Self-reported FS in the past: 54.0% Self-reported CS in the past: 49.2%	Adults aged ≥ 18 years	For FOBT: Physician recommendation: Adj OR = 3.71 (95%CI = 1.11, 12.46) For FS: Physician recommendation: Adj OR = 9.10 (95%CI = 1.87, 44.21) For CS: Physician recommendation: Adj OR = 9.52 (95%CI = 1.56, 58.82)	For FOBT: Acculturation; Risk perception. For FS: Acculturation; Risk perception. For CS: Acculturation; Risk perception.	6
Tu *et al.* [32]	2006	USA	Interventional	210	Medical record of FOBT: 48.5%	Adults aged ≥ 50 years	No significant factors other than the intervention	Age; Gender; Language; Insurance; Prior FOBT.	7
Wang *et al.* [26]	2006	USA	Cross-sectional	433	Self-reported CRC screening according to US guideline: 57%	Women aged ≥50 years	Physician recommendation: Adj OR = 3.44 (95%CI = 2.25, 5.28) Symptoms: Adj OR = 1.74 (95%CI = 1.10, 2.73) Thoughts about getting CRC: Adj OR = 2.79 (95%CI = 1.63, 4.77) Cultural views: Adj OR = 0.97 (95%CI = 0.95, 0.99)	All variables are significant in the multivariate analysis.	6
Yip *et al.* [31]	2006	USA	Cross-sectional	383	Medical record of CRC screening : 40%	Adults aged 50–78 years	No significant factor	Age; Gender; Insurance status; Language.	6
Yu *et al.* [35]	2001	USA	Cross-sectional	664	Self-reported DRE in the past: 14.1% Self-reported FOBT in the past: 8.5%	Random sample of adults aged 40–69 years	For DRE: Education ≥ 12 years: Adj OR = 2.38 (95%CI = 1.47, 3.84) For FOBT: Age < 55: Adj OR = 0.46 (95%CI = 0.29, 0.72)	For DRE: Age; Gender; Usual source of care; Cancer warning signs; Length of residency in US. For FOBT: Gender; Education; Usual source of care; Cancer warning signs; Length of residence in US.	6

* Estimates of effect sizes of the factors were not available in the study; FOBT = fecal occult blood testing, FIT = faecal immunochemical test, CS = colonoscopy, FS = flexible sigmoidoscopy, DRE = digital rectal examination.
